# WRKY36–PIL15 Transcription Factor Complex Negatively Regulates Sheath Blight Resistance and Seed Development in Rice

**DOI:** 10.3390/plants14040518

**Published:** 2025-02-08

**Authors:** Siting Wang, Qian Sun, Shuo Yang, Huan Chen, Depeng Yuan, Changxi Gan, Haixia Chen, Yongxi Zhi, Hongyao Zhu, Yue Gao, Xiaofeng Zhu, Yuanhu Xuan

**Affiliations:** 1 College of Plant Protection, Shenyang Agricultural University, Shenyang 110866, China; 2021200147@stu.syau.edu.cn (S.W.); sunqian5328@syau.edu.cn (Q.S.); 2021200139@stu.syau.edu.cn (S.Y.); ichenhuan0321@outlook.com (H.C.); 2023200160@stu.syau.edu.cn (H.Z.); gaoyue@syau.edu.cn (Y.G.); 2 State Key Laboratory of Elemento-Organic Chemistry and Department of Plant Protection, National Pesticide Engineering Research Center (Tianjin), Nankai University, Tianjin 300071, China; dpyuan@outlook.com; 3 Zhengzhou Lvyeyuan Agricultural Technology Co., Ltd., Zhengzhou 450016, China; lyygcx1688@163.com (C.G.); 13523088205@163.com (H.C.); yongxizhi@163.com (Y.Z.)

**Keywords:** *SWEET11*, *miR530*, *Rhizoctonia solani*, AOS2, WRKY53

## Abstract

Sheath blight (ShB) causes severe yield loss in rice. Previously, we demonstrated that the *sugar will eventually be exported and the transporter 11* (*SWEET11*) mutation significantly improved rice resistance to ShB, but it caused severe defects in seed development. The present study found that WRKY36 and PIL15 directly activate *SWEET11* to negatively regulate ShB. Interestingly, WRKY36 interacted with PIL15, WRKY36 and PIL15 directly activates *miR530* to negatively regulate seed development. WRKY36 interacted with a key BR signaling transcription factor WRKY53. AOS2 is an effector protein from *Rhizoctonia solani* (*R. solani*) that interacts with WRKY53. Interestingly, AOS2 also interacts with WRKY36 and PIL15 to activate *SWEET11* for sugar nutrition for *R. solani*. These data collectively suggest that WRKY36–PIL15 negatively regulates ShB resistance and seed development via the activation of *SWEET11* and *miR530*, respectively. In addition, WRKY36 and PIL15 are the partners of the effector protein AOS2 by which *R. solani* hijacks sugar nutrition from rice.

## 1. Introduction

Host plants have evolved disease resistance strategies to resist pathogens during their interaction. Sheath blight (ShB) in rice, caused by *Rhizoctonia solani* (*R. solani*), is the one of the most important diseases in rice, which can cause up to 50% yield loss [1]. Previous studies revealed that the resistance against ShB is controlled by multiple genes. At present, some of the quantitative trait locus (QTL)-linked genes have been cloned and functionally verified [2,3]. Phytohormone signals play an important role in the process of resistance of rice to ShB. Jasmonic acid (JA), salicylic acid (SA), and auxin signals regulate the resistance of rice to ShB [4,5]. Our previous study confirmed that ethylene and brassinosteroid (BR) signals positively and negatively regulate the resistance of rice to ShB, respectively [6]. WRKY transcription factors (TFs) are widely involved in the resistance of rice to ShB [7]. OsWRKY53 negatively regulates ShB resistance by activating *SWEET2a* [8]. In addition, SlWRKY36 and SlWRKY51 act as positive regulatory factors for salt stress tolerance, regulating tomato salt tolerance [9]. A recent study reported that the effector protein AOS2 from *R. solani* interacts with the rice TFs OsWRKY53 and OsGT1 and forms a TF complex to activate the expression of *OsSWEET2a* and *OsSWEET3a*, thereby promoting the pathogenesis of ShB [10].

The results of the genome-wide association study revealed that the natural variation of *ZmFBL41* in maize affected the interaction between ZmFBL41 and ZmCAD, further affecting the host resistance [11]. Our previous study confirmed that *OsLPA1* overexpression improves the resistance of rice to ShB [12]. A further study reported that OsLPA1 regulates the resistance of rice to ShB through interaction with OsKLP [13]. OsPhyB and OsLAZY1 negatively regulate the resistance of rice to ShB [14]. OsDEP1 interacts with OsLPA1 and inhibits the transcriptional activation of OsLPA1 downstream genes, thereby inhibiting the resistance of rice to ShB [15]. In addition, the protein phosphatase 2A catalytic subunit (PP2A-1) positively regulates the resistance of rice to ShB [16].

The interaction between plant pathogens and hosts is a complex and prolonged process. Extensive breeding practices have proven that improving host disease resistance often reduces crop yield, which is a trade-off between plant development and disease resistance [17,18]. Therefore, effective disease prevention and control, particularly exploring important genes that can simultaneously increase yield and disease resistance, is of great significance for ensuring national food security. The SA and JA pathways typically interact in an antagonistic manner and interact with the pathways of other plant hormones such as abscisic acid (ABA), gibberellic acid (GA), BR, and auxin to jointly regulate the trade-off between growth and immunity [19,20]. TFs are important factors that balance plant growth and immunity [21]. BZR1 blocks PTI signaling by interacting with different WRKYs or via transcriptional regulation with different WRKYs in *Arabidopsis* [18]. WRKY forms a transcription protein complex with MVQ (MPK3/6-targeted VQPs) protein, controlling the transcription of defense genes [22]. Our previous study reported that *PIL15* negatively regulates rice resistance to ShB [23]. In addition, PIL15 activated *miR530*, a negative regulator of grain size to negatively regulate grain size [24]. PIL15 directly targets *OsPUP7*, affecting the transport of cytokinin, thereby affecting cell division and subsequent particle size [25]. The immune receptor NLR and cell-wall-associated kinase (WAK) play a dual role in balancing immunity and production. The two *NLR* genes at the *Pigm* locus not only confer resistance to *Aspergillus oryzae* but also increase yield [26]. *Xa4* encodes a WAK and confers race-specific persistent resistance to *Xoo* without losing production potential [27].

SWEET is a family of sugar transporters. It has the function of bidirectional transport of sugar substances [28] and is involved in regulating plant–pathogen interactions [29]. Most studies have reported that activating the expression of the *SWEET*s is beneficial for pathogenic bacteria to hijack carbohydrate nutrients from plants to promote their growth [28,30]. In addition, some studies have reported that inhibiting the expression of *SWEET* can reduce the sugar content in the extracellular space and inhibit the growth of pathogenic bacteria [31]. *OsSWEET11/Xa13/Os8N3*, *OsSWEET13/Xa25*, and *OsSWEET14/Os11N3* are important genes in rice regulating resistance to *Xanthomonas oryzae* pv. *oryzae* [32,33].

Our previous study reported that *OsSWEET11* is significantly induced by *R. solani* infection, and *sweet11* mutants are less susceptible to ShB [34]. However, in *sweet11* mutants, the sucrose concentration in the embryo sacs significantly decreased and led to defective grain filling, which resulted in significantly lower 100-grain weight in *sweet11* mutants than in wild-type plants [35]. In addition, we previously demonstrated Rubisco promoter expressing sucrose transport activity defected SWEET11 mutant protein increased ShB resistance and maintained normal grain filling [34]. Tissue-specific activation of DOF11 promoted rice resistance to ShB via the activation of *SWEET14* without yield loss [31]. This suggested that tissue-specific regulation of *SWEET* expression might achieve the goal of improvement in ShB resistance without developmental defects in rice. Numerous studies have reported that WRKY TFs are widely involved in the resistance of rice to ShB [8,36]. In addition, our previous study reported that *PIL15* repressors and overexpressors were less and more susceptible to ShB, respectively [23]. However, the regulation of *SWEETs* by TFs to modulate plant defense has not been studied in detail.

In this study, we observed that the WRKY36 and PIL15 activate *SWEET11*. Further, WRKY36 interacted with PIL15 to activate *SWEET11*, which negatively regulated rice resistance to ShB. In addition, WRKY36–PIL15 activated *miR530* to negatively regulate grain size. *R. solani* effector protein AOS2 interacted with WRKY36 and PIL15 to activate *SWEET11* for sugar efflux, via which *R. solani* activated *SWEET11* expression as well as subsequent sugar efflux. Our study revealed the mechanism by which *R. solani* activated *SWEET11* for sugar efflux and identified WRKY36 and PIL15 as the potential molecular targets in ShB-resistant breeding.

## 2. Results

### 2.1. WRKY36 and PIL15 Negatively Regulate Resistance of Rice to ShB

**Figure 1:**
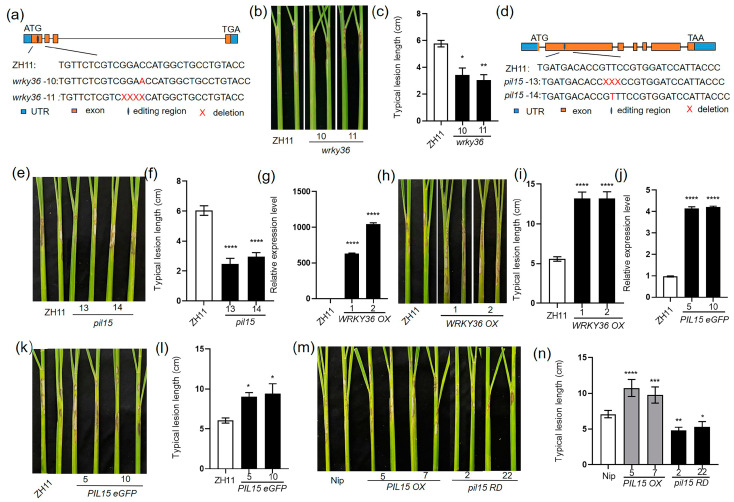
Response of *WRKY36* and *PIL15* to *Rhizoctonia solani* AG1-1A. (**a**,**d**) Mutation site sequence information of *WRKY36* and *PIL15* genome-editing mutants generated using CRISPR/Cas9. (**b**) WT and *wrky36* mutants were inoculated with *R. solani*. (**c**) The length of lesions on the leaf sheath surface was measured. (**e**) WT and *pil15* mutants were inoculated with *R. solani*. (**f**) The length of lesions on the leaf sheath surface was measured. (**g**) The expression of *WRKY36* in WT and *WRKY36 OXs* was examined using RT-qPCR. (**h**) WT and *WRKY36 OXs* were inoculated with *R. solani*. (**i**) The length of lesions on the leaf sheath surface was measured. (**j**) The expression of *PIL15* in WT and *PIL15 eGFP OXs* was examined using RT-qPCR. (**k**) WT and *PIL15 eGFP OXs* were inoculated with *R. solani*. (**l**) The length of lesions on the leaf sheath surface was measured. (**m**) WT, *PIL15 OXs* and *pil15 RDs* were inoculated with *R. solani*. (**n**) The length of lesion on the leaf sheath surface was measured. Statistical differences between groups were analyzed using the Student’s *t*-test (* *p* < 0.05; ** *p* < 0.01; *** *p* < 0.001; **** *p* < 0.0001).

To isolate the transcription factor that might specifically regulate *SWEET11*, the yeast-one hybrid screening was performed using 2.0 kb of *SWEET11* promoter as the bait. The results identified 15 putative transcriptional regulators including WRKY36 and PIL15. Previously, WRKYs and PIL15 were reported to regulate rice ShB resistance [7,23]; therefore, WRKY36 and PIL15 were further analyzed in relation to their regulation on *SWEET11* transcription. The *R. solani*-dependent expression patterns revealed that *PIL15* was significantly induced, whereas *WRKY36* expression had no significant difference in *R. solani* infection (Figure S1). Therefore, the functions of *WRKY36* and *PIL15* in the resistance of rice to ShB were further investigated. Via genome editing, *wrky36* and *pil15* mutants were generated. *wrky36-10* and *wrky36-11* mutants have 1-bp insertion and 4-bp deletion at the 1st exon, respectively (Figure 1a). After *R. solani* inoculation, *wrky36* mutants were less susceptible to ShB than the wild-type plants (Figure 1b,c). *pil15-13* and *pil15-14* mutants have 3-bp deletion and 1-bp insertion at the 2nd exon, respectively (Figure 1d). After *R. solani* inoculation, *pil15* mutants were less susceptible to ShB than the wild-type plants (Figure 1e,f).

Since *wrky36* and *pil15* mutants were less susceptible to ShB, the mechanisms of *WRKY36* and *PIL15* in ShB resistance were further examined. To further evaluate the defense mechanism, plants overexpressing *WRKY36* and *PIL15* were tested. *WRKY36* expression level was clearly higher in *WRKY36 OX 1* and *OX 2* than in wild-type plants (Figure 1g). After *R. solani* inoculation, *WRKY36 OX* plants were more susceptible to ShB than the wild-type plants (Figure 1h,i). *PIL15* expression level was clearly higher in *PIL15 eGFP OX 5* and *OX 10* than in wild-type plants (Figure 1j). After *R. solani* inoculation, *PIL15 eGFP OX* plants were more susceptible to ShB than the wild-type plants (Figure 1k,l). Similarly, the *PIL15* overexpression (*PIL15 OX*) and repressor (*PIL15 RD*) lines were examined [37]. *PIL15 RDs* were less susceptible to ShB than the wild-type plants, whereas *PIL15 OXs* were more susceptible to ShB than the wild-type plants (Figure 1m,n).

### 2.2. WRKY36 Interacts with PIL15 to Activate SWEET11

**Figure 2:**
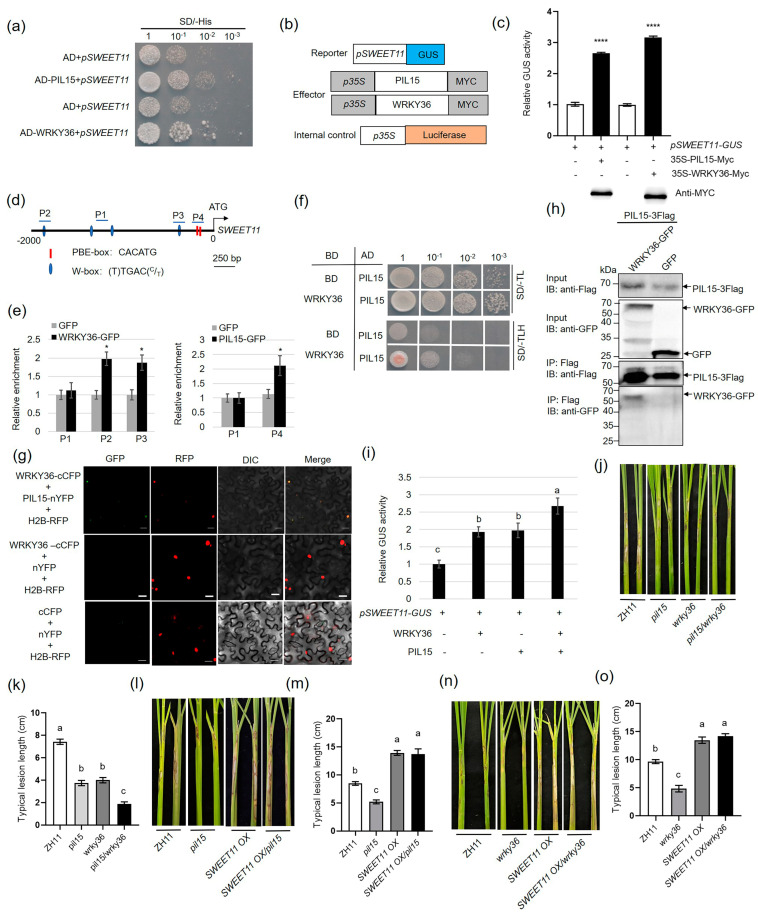
Confirmation of activation of *SWEET11* by WRKY36 or PIL15, and the interaction between WRKY36 and PIL15. (**a**) Yeast-one hybrid assay of the binding of PIL15 or WRKY36 to the *SWEET11* promoter. (**b**) Reporter, effector, and internal control vector diagram. (**c**) Transactivation assays verified that WRKY36 and PIL15 activated *SWEET11* expression. (**d**) The putative W-box and PBE-box elements in the 2.0-kb region of the *SWEET11* promoter. (**e**) ChIP-qPCR assay showing that WRKY36 and PIL15 directly bind to the W-box and PBE-box motif in the *SWEET11* promoter, respectively. The promoter fragment of *SWEET11* containing the W-box motif (P2, P3) was enriched in the ChIP-qPCR analysis rather than in the negative control (P1). Similarly, the promoter fragment of *SWEET11* containing the PBE-box motif (P4) was enriched in the ChIP-qPCR analysis rather than in the negative control (P1). (**f**) Yeast-two hybrid assay verified the interaction between PIL15 and WRKY36. (**g**) BiFC assay verified the interaction between PIL15 and WRKY36 in *N. benthamiana* leaves. Co-transformation of PIL15-nYFP and WRKY36–cCFP led to the reconstitution of GFP signal, whereas no signal was detected when WRKY36–cCFP and nYFP were co-expressed. Co-transformation of cCFP and nYFP was used as negative control. (**h**) Co-IP assays indicated the interaction between PIL15 and WRKY36 in plants. PIL15-3xFlag and WRKY36–GFP were co-expressed in *N. benthamiana* leaves. The co-expression of PIL15-3xFlag and GFP was used as a negative control. (**i**) The transient assay showed that WRKY36 and PIL15 both activated *SWEET11* expression in an additive manner. The promoter of the *SWEET11*-driven reporter gene was co-expressed with WRKY36 or PIL15 individually or together. (**j**) WT, *pil15*, *wrky36*, *wrky36/pil15* plants were inoculated with *R. solani*. (**k**) The length of lesions on the leaf sheath was measured. (**l**) WT, *pil15*, *SWEET11 OX*, *SWEET11 OX/pil15* plants were inoculated with *R. solani*. (**m**) The length of lesions on the leaf sheath was measured. (**n**) WT, *wrky36*, *SWEET11 OX*, *SWEET11 OX/wrky36* plants were inoculated with *R. solani*. (**o**) The length of lesions on the leaf sheath was measured. Statistical differences between groups were analyzed using the Student’s *t*-test (* *p* < 0.05; **** *p* < 0.0001). Different letters above the bars indicate significant differences at *p* < 0.05.

Yeast-one hybrid (Y1H) assay results revealed that WRKY36 or PIL15 activates the 2.0-kb *SWEET11* promoter (Figure 2a). To verify the Y1H assay results, a transient assay was performed in rice protoplast cells. The results revealed that the co-expression of WRK36-Myc or PIL15-Myc with 2.0 kb of *pSWEET11-GUS* significantly activated the *GUS* expression level than the expression of *pSWEET11-GUS* alone. Western blot analysis was performed to detect the successful expression of WRK36-Myc or PIL15-Myc in the rice protoplast using an anti-Myc antibody (Figure 2b,c). Further, the *SWEET11* promoter sequences were analyzed. The results indicated that the *SWEET11* promoter region contains (T)TGAC(^C^/_T_) W-box (WRKY-binding) and (CACATG) PBE-box (PIL-binding) motifs (Figure 2d). Chromatin immunoprecipitation (ChIP) assay using WRKY36–GFP and PIL15–GFP plant calli as well as anti-GFP antibody revealed that WRKY36 bound to the P2 and P3 regions harboring W-box motif, whereas PIL15 bound to P4 with the enrichment of PBE-box motifs (Figure 2e).

Since both WRKY36 and PIL15 activate *SWEET11*, the interaction between WRKY36 and PIL15 was investigated. Yeast-two hybrid (Y2H) assay revealed that WRKY36 interacts with PIL15 (Figure 2f). Bimolecular fluorescence complementation (BiFC) assay with WRKY36 and PIL15 expression in *Nicotiana benthamiana* leaves revealed that WRKY36 interacts with PIL15 in the nucleus (Figure 2g). Co-immunoprecipitation (Co-IP) assay was performed by co-expressing WRKY36–GFP and PIL15-3xFlag or GFP and PIL15-3xFlag. Subsequent immunoprecipitation via anti-Flag antibody as well as Western blot analysis indicated that WRKY36–GFP interacts with PIL15-3xFlag (Figure 2h).

Furthermore, the function of WRKY36–PIL15 in *SWEET11* activation was analyzed. The transient assay results revealed that WRKY36 or PIL15 expression activated *pSWEET11*, whereas co-expression of WRKY36 and PIL15 exhibited a stronger transcription activation activity than the expression of WRKY36 or PIL15 alone (Figure 2i). Further, *wrky36/pil15* double mutants and *SWEET11 OX/pil15* or *SWEET11 OX/wrky36* plants were generated. After *R. solani* inoculation, *pil15 and wrky36* single mutants were less and more susceptible to diseases than wild-type and *wrky36/pil15* double mutants, respectively (Figure 2j,k). Inoculation of *R. solani* showed that *SWEET11 OX* and *SWEET11 OX/pil15* plants exhibited similar lesion length, and *SWEET11 OX* and *SWEET11 OX/pil15* plants were more susceptible than *pil15* (Figure 2l,m). Similarly, inoculation of *R. solani* identified that *SWEET11 OX* and *SWEET11 OX/wrky36* plants developed similar lesion length, and *SWEET11 OX* and *SWEET11 OX/wrky36* plants were more susceptible than *wrky36* (Figure 2n,o). In addition, the expression levels of *SWEET11* in *wrky36/pil15* double mutants and *SWEET11 OX/pil15* or *SWEET11 OX/wrky36* leaf sheaths were also detected. The results showed that the expression level of *SWEET11* in *pil15 and wrky36* single mutants were lower than that in the wild-type and higher than that in *wrky36/pil15* double mutants (Figure S2a).

The expression levels of *SWEET11* in *SWEET11 OX* and *SWEET11 OX/pil15* leaf sheaths were similar, and *SWEET11 OX* and *SWEET11 OX/pil15* plants were higher than *pil15* and wild-type plants (Figure S2b). Similarly, the expression levels of *SWEET11* in *SWEET11 OX* and *SWEET11 OX/wrky36* leaf sheaths were similar, and *SWEET11 OX* and *SWEET11 OX/wrky36* plants were higher than *wrky36* and wild-type plants (Figure S2c). Meanwhile, the expression levels of *SWEET11* in *PIL15 eGFP OX* and *WRKY36 OX* leaf sheaths were also detected. The results showed that the expression level of *SWEET11* in *PIL15 eGFP OX* and *WRKY36 OX* plants was higher than that in wild-type plants (Figure S2d).

### 2.3. WRKY36 and PIL15 Negatively Regulate Seed Development

**Figure 3:**
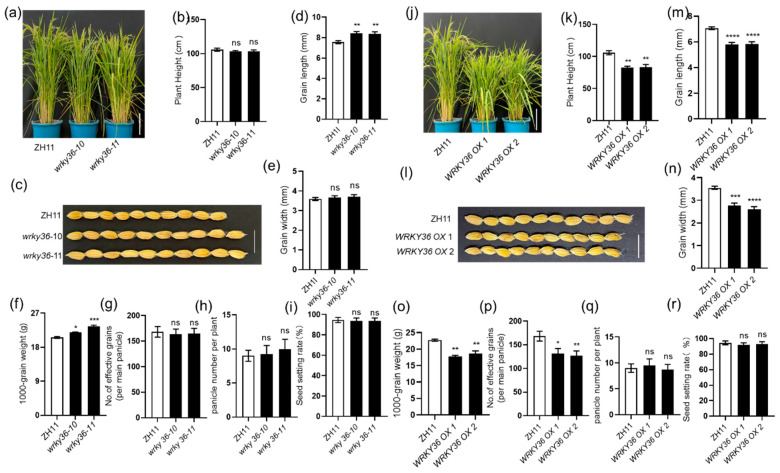
WRKY36 regulates seed development. (**a**) Phenotypes of 3-month-old *wrky36* mutants and WT plants. Scale bar = 20 cm. (**b**) The height of plants was measured. (**c**) Grains with hulls from *wrky36* and WT. Scale bar = 1 cm. (**d**) Seed length of *wrky36* and WT. (**e**) Seed width of *wrky36* and WT. (**f**) The 1000-grain weight of *wrky36* and WT. (**g**) Number of effective grains per panicle in *wrky36* and WT. (**h**) Panicle number per plant of *wrky36* and WT. (**i**) The seed setting rate (%) of *wrky36* and WT. (**j**) Phenotypes of 3-month-old *WRKY36 OXs* and WT plants. Scale bar = 20 cm. (**k**) The height of plants was measured. (**l**) Grains with hulls from *WRKY36 OXs* and WT. Scale bar = 1 cm. (**m**) Seed length of *WRKY36 OXs* and WT. (**n**) Seed width of *WRKY36 OXs* and WT. (**o**) The 1000-grain weight of *WRKY36 OXs* and WT. (**p**) Number of effective grains per panicle in *WRKY36 OX* and WT. (**q**) Panicle number per plant of *WRKY36 OX* and WT. (**r**) The seed setting rate (%) of *WRKY36 OX* and WT. The data in (**b**,**d**–**i**,**k**,**m**–**r**) are presented as the mean ± SE (**b**,**g**–**i**,**k**,**p**–**r**, n = 20 plants; **c**–**f** and **l**–**o**, n > 100 seeds; **f**,**o**, n = 3 replicates). Statistical differences between groups were analyzed using the Student’s *t*-test (* *p* < 0.05; ** *p* < 0.01; *** *p* < 0.001; **** *p* < 0.0001; ns indicates nonsignificant).

WRKY36–PIL15 activates *SWEET11* to regulate resistance of rice to ShB, and *sweet11* mutants exhibited defective seed development. Therefore, the seed development of *WRKY36* and *PIL15* mutants or overexpression plants was assessed. First, the plant height of *wrky36* mutants and wild-type plants was similar (Figure 3a,b). Interestingly, *wrky36* mutants developed relatively longer seeds than wild-type plants (Figure 3c,d); however, the width of seeds of *wrky36* mutants and wild-type plants were similar (Figure 3e). Additionally, 1000-grain weight of *wrky36* mutants was higher than that of wild-type plants (Figure 3f). However, there is no difference in the seed setting ration, panicle number per plant, and seed number per panicle between *wrky36* mutants and wild-type plants (Figure 3g–i). The height of *WRKY36 OX* plants was significantly shorter than that of wild-type plants (Figure 3j,k). *WRKY36 OX* plants developed smaller seeds than wild-type plants. The seed length of *WRKY36 OX* plants was shorter, whereas the width was narrower than wild-type plants (Figure 3l–n). This developmental feature resulted in lower 1000-grain weight in *WRKY36 OX* than in wild-type plants (Figure 3o). *WRKY36 OX* plants have fewer seed numbers per panicle compared to wild-type plants (Figure 3p). However, there is no difference in the seed setting ration and panicle number per plant between *WRKY36 OX* and wild-type plants (Figure 3q,r).

**Figure 4:**
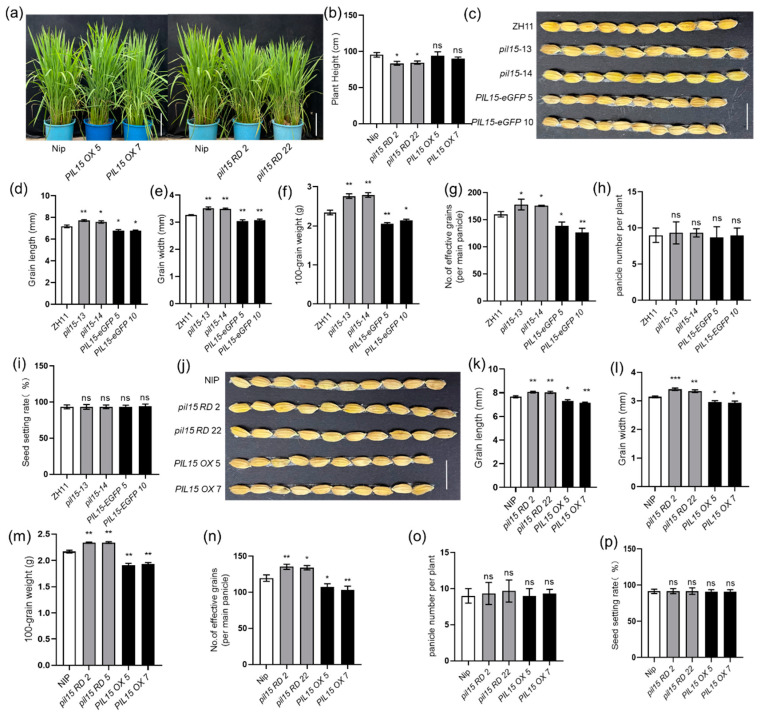
PIL15 regulates seed development. (**a**) Phenotypes of 3-month-old *PIL15 RD* mutants, *PIL15 OXs* and WT plants. Scale bar = 20 cm. (**b**) The height of plants was measured. (**c**) Grains with hulls from *pil15* mutants, *PIL15 eGFP OXs*, and WT. Scale bar = 1 cm. (**d**) Seed length of *pil15* mutants, *PIL15 eGFP OXs*, and WT. (**e**) Seed width of *pil15* mutants, *PIL15 eGFP OXs*, and WT. (**f**) The 100-grain weight of *pil15* mutants, *PIL15 eGFP OXs*, and WT. (**g**) Number of effective grains per panicle in *pil15* mutants, *PIL15 eGFP OXs*, and WT. (**h**) Panicle number per plant of *pil15* mutants, *PIL15 eGFP OXs*, and WT. (**i**) The seed setting rate (%) of *pil15* mutants, *PIL15 eGFP OXs*, and WT. (**j**) Grains with hulls from *pil15 RDs*, *PIL15 OXs*, and WT. Scale bar = 1 cm. (**k**) Seed length of *pil15 RDs*, *PIL15 OXs*, and WT. (**l**) Seed width of *pil15 RDs*, *PIL15 OXs*, and WT. (**m**) The 100-grain weight of *pil15 RDs*, *PIL15 OXs*, and WT. (**n**) Number of effective grains per panicle in *pil15 RDs*, *PIL15 OXs*, and WT. (**o**) Panicle number per plant of *pil15 RDs*, *PIL15 OXs*, and WT. (**p**) The seed setting rate (%) of *pil15 RDs*, *PIL15 OXs*, and WT. The data in (**b**–**i**,**k**–**p**) are presented as the mean ± SE (**b**,**g**–**i**,**n**–**p**, n = 20 plants; **c**–**f**,**j**–**m**, n > 100 seeds; **f**,**m**, n = 3 replicates). Statistical differences between groups were analyzed using the Student’s *t*-test (* *p* < 0.05; ** *p* < 0.01; *** *p* < 0.001; ns indicates nonsignificant).

First, the plant heights of *pil15 RD* mutants, *PIL15 OX* plants and wild-type plants were detected (Figure 4a,b). *pil15 RD* mutants have a shorter plant height than the wild-type plants, while *PIL15 OX* plants show no difference. Next, the function of *PIL15* in rice seed development was analyzed. The seed length and width of *pil15* mutants were relatively longer, whereas those of *PIL15 eGFP OX plants* were relatively shorter than those of wild-type plants (Figure 4c–e). These developmental differences resulted in higher 100-grain weight of *pil15* mutants and lower 100-grain weight of *PIL15 eGFP OX* than that of wild-type plants (Figure 4f). In addition, compared with the wild-type plants, *PIL15 eGFP OX* plants have fewer seed number per panicle, while *pil15* mutants has more seed number per panicle (Figure 4g). However, the seed setting ration and panicle number per plant of *pil15* mutants, *PIL15 eGFP OX* and wild-type plants were similar (Figure 4h,i). Similarly, *PIL15 OXs* and *PIL15 RDs* were examined. *PIL15 RDs* developed larger seeds, whereas *PIL15 OXs* developed smaller seeds than wild-type plants (Figure 4j–l), and the 100-grain weight was higher in *PIL15 RDs* but lower in *PIL15 OXs* than in wild-type plants (Figure 4m). In addition, compared with wild-type plants, *PIL15 OXs* have fewer seed numbers per panicle, while *PIL15 RDs* have more seed numbers per panicle (Figure 4n). However, the seed setting ration and panicle numbers per plant of *pil15 RDs*, *PIL15 OXs* and wild-type plants were similar (Figure 4o,p).

### 2.4. WRKY36–PIL15 Directly Activates miR530

**Figure 5:**
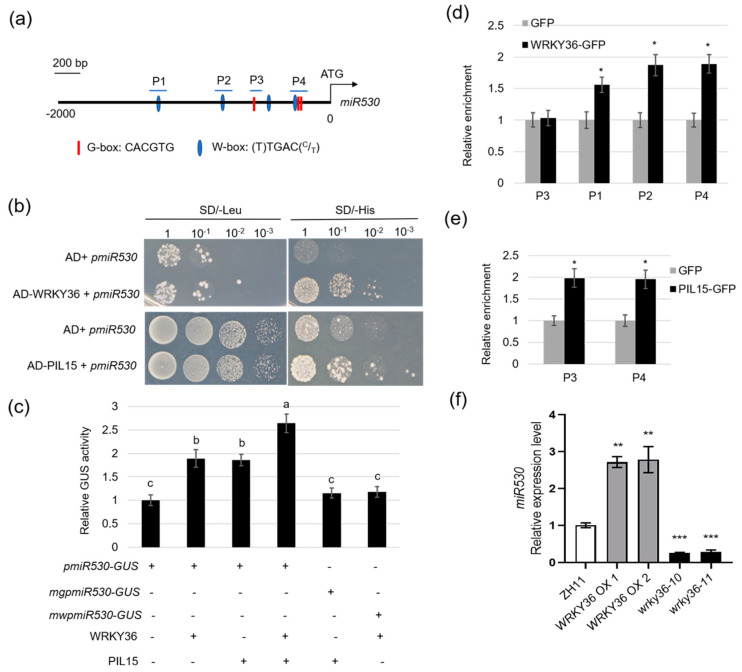
Confirmation of activation of *miR530* by WRKY36–PIL15. (**a**) Putative W-box and G-box elements in the 2.0-kb region of the *miR530* promoter. (**b**) Yeast-one hybrid assay of the binding of PIL15 and WRKY36 to the *miR530* promoter. (**c**) The transient assay showed that WRKY36 and PIL15 both activated *miR530* expression in an additive manner. WRKY36 and PIL15 cannot activate *miR530* transcription with W-box and G-box site mutations in the *miR530* promoter (*mwpmiR530* and *mgpmiR530*). (**d**) ChIP-qPCR assay showing that WRKY36 directly bind to the W-box motif in the *miR530* promoter. The promoter fragment of *miR530* containing the W-box motif (P1, P2, and P4) was enriched in the ChIP-qPCR analysis rather than in the negative control (P3). (**e**) ChIP-qPCR assay showing that PIL15 directly bind to the G-box motif in the *miR530* promoter. The promoter fragment of *miR530* containing the G-box motif (P3, P4) was enriched in the ChIP-qPCR analysis. (**f**) Expression of *miR530* (*miR530* precursor) in *wkry36*, *WRKY36 OX* and WT plants. *Ubiquitin* was used as the internal control for RT-qPCR. The data in (**c**–**f**) are presented as the mean ± SE (n = 3). Statistical differences between groups were analyzed using the Student’s *t*-test (* *p* < 0.05; ** *p* < 0.01; *** *p* < 0.001). Different letters above the bars indicate significant differences at *p* < 0.05.

WRKY36 and PIL15 activate *SWEET11* to regulate the resistance of rice to ShB, and *sweet11* mutants exhibited defective seed development; therefore, the expression levels of *SWEET11* in the seeds of *WRKY36* and *PIL15* mutants were examined. The results showed that there was no difference in the expression level of *SWEET11* between *WRKY36* and *PIL15* mutants compared to wild-type plants (Figure S3). It is proved that WRKY36 and PIL15 might regulate the expression of *SWEET11* in leaf sheaths, but not in the seeds. A previous study revealed that PIL15 activates *miR530* to negatively regulate grain size [24]. To analyze whether WRKY36 also activates *miR530*, *miR530* promoter sequences were analyzed. The W-box motif was observed within 2.0 kb of *miR530* promoter region [38,39] (Figure 5a). The Y1H assay results indicated that both WRKY36 and PIL15 activate *pmiR530* (Figure 5b). To verify the results of Y1H assay, the transient assay was performed. In the protoplast cells, WRKY36 or PIL15 activated *pmiR530* but not W-box (*mwpmiR530*)- or G-box (*mgpmiR530*)-mutated *pmiR530*. Additionally, co-expression of WRKY36 and PIL15 exhibited an additive effect on the activation of *pmiR530* (Figure 5c). ChIP-qPCR results further confirmed that WRKY36 and PIL15 bound to the *miR530* promoter (Figure 5d,e). To analyze *miR530* expression levels in *wkry36* mutants and *WRKY36 OX* plants, the *miR530* expression levels were assessed in the developing caryopsis of *wrky36* mutants, *WRKY36 OX*, and wild-type plants. The RT-qPCR results revealed that the *miR530* expression level was significantly lower in *wrky36* mutants but higher in *WRKY36 OX* plants than in wild-type plants (Figure 5f).

### 2.5. AOS2 Interacts with WRKY36 and PIL15 to Activate SWEET11

**Figure 6:**
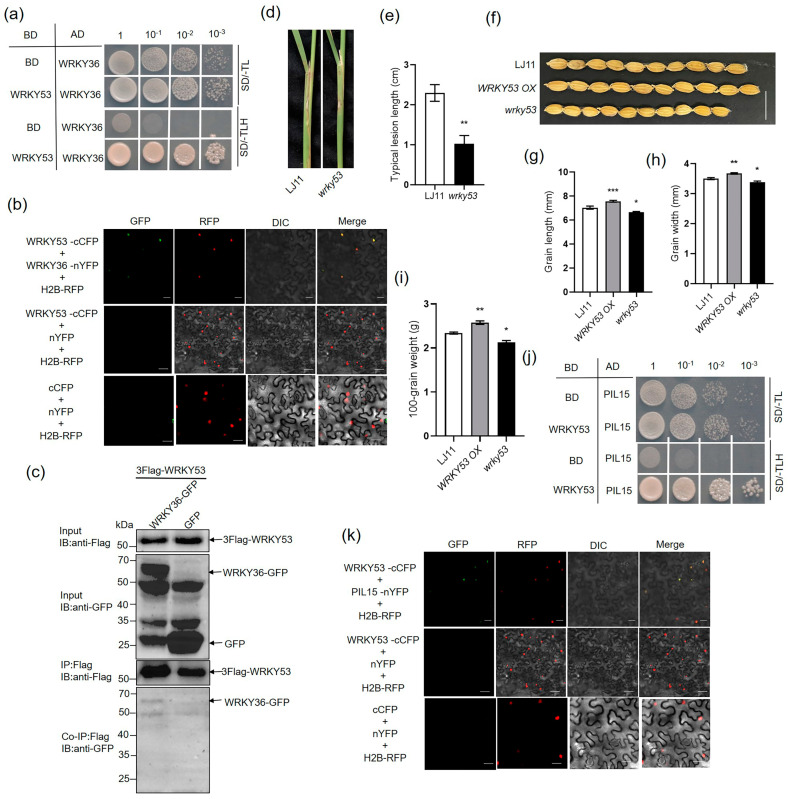
Confirmation of the interaction between WRKY36 and WRKY53. (**a**) Yeast-two hybrid assay verified the interaction between WRKY53 and WRKY36. (**b**) BiFC assay indicated the interaction between WRKY53 and WRKY36 in *N. benthamiana* leaves. Co-transformation of WRKY36–nYFP and WRKY53–cCFP led to the reconstitution of GFP signal, whereas no signal was detected when WRKY53–cCFP and nYFP were co-expressed. Co-transformation of cCFP and nYFP was used as negative control. (**c**) Co-IP assays indicated the interaction between WRKY53 and WRKY36 in plants. 3xFlag-WRKY53 and WRKY36–GFP were co-expressed in *N. benthamiana* leaves. The co-expression of 3xFlag-WRKY53 and GFP was used as a negative control. (**d**) WT and *wrky53* mutants were inoculated with *R. solani*. (**e**) The length of lesions on the leaf sheath surface was measured. (**d**) Six leaf sheaths from each line were analyzed, and the experiments were repeated three times. The data in (**e**) are presented as the mean ± SE (n > 10). (**f**) Grains with hulls from *wrky53*, *WRKY53 OX*, and WT. Scale bar = 1 cm. (**g**) Seed length of *wrky53*, *WRKY53 OX*, and WT. (**h**) Seed width of *wrky53*, *WRKY53 OX*, and WT. (**i**) The 100-grain weight of *wrky53*, *WRKY53 OX*, and WT. The data in (**g**–**i**) are presented as the mean ± SE. (**f**–**h**, n > 100 seeds; **i**, n = 3 replicates). (**j**) Yeast-two hybrid assay verified the interaction between WRKY53 and PIL15. (**k**) BiFC assay indicated the interaction between WRKY53 and PIL15 in *N. benthamiana* leaves. Co-transformation of PIL15-nYFP and WRKY53-cCFP led to the reconstitution of GFP signal, whereas no signal was detected when WRKY53-cCFP and nYFP were co-expressed. Co-transformation of cCFP and nYFP was used as negative control. Statistical differences between groups were analyzed using the Student’s *t*-test (* *p* < 0.05; ** *p* < 0.01; *** *p* < 0.001).

Plants overexpressing *WRKY36* exhibited an enlarged lamina joint angle (Figure 3g), a typical BR signaling phenotype in rice [40]. Further, Y2H assay revealed that WRKY36 interacts with WRKY53 (Figure 6a), the key BR signaling TF [41]. BiFC assay results confirmed that WRKY36 and WRKY53 interact in the nucleus of tobacco cells (Figure 6b). In addition, Co-IP assay results indicated that 3xFlag-WRKY53 interacts with WRKY36–GFP but not with GFP, indicating that WRKY36 interacts with WRKY53 (Figure 6c). After *R. solani* inoculation, *wrky53* mutants were less susceptible to the ShB than wild-type plants (Figure 6d,e). However, *wrky53* mutant seeds were smaller, and *WRKY53 OX* seeds were bigger than wild-type seeds, which was different from the results of *wrky36* mutants (Figure 6f–i) [41]. In addition, the interaction between PIL15 and WRKY53 was assessed. The results of the Y2H assay revealed that WRKY53 interacts with PIL15 (Figure 6j). The BiFC results confirmed that WRKY53 interacts with PIL15 in the nucleus of tobacco cells (Figure 6k). However, unlike WRKY36 and PIL15, WRKY53 did not directly activate the *SWEET11* promoter (Figure S4). The results indicated that WRKY53 might play a similar function as that of WRKY36 and PIL15 in the resistance of rice to ShB but not in grain development.

**Figure 7:**
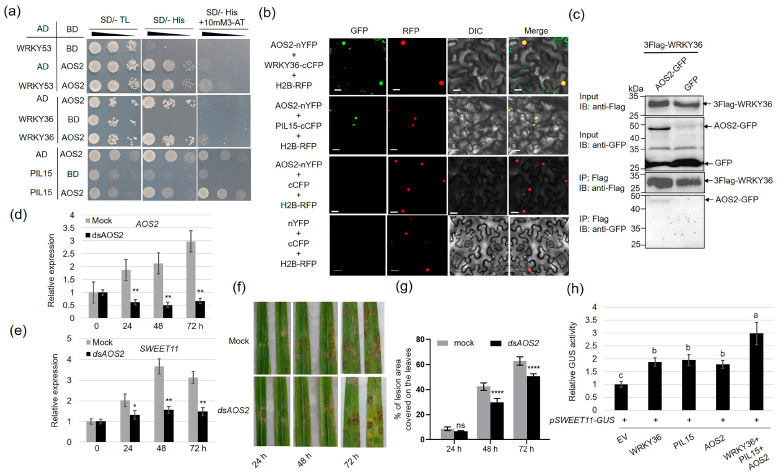
AOS2–WRKY36–PIL15 transcriptional complex activates *SWEET11.* (**a**) Yeast-two hybrid assay verified the interaction of WRKY53, PIL15, and WRKY36 with AOS2. (**b**) BiFC assay indicated the interaction of WRKY36 and PIL15 with AOS2 in *N. benthamiana* leaves. Co-transformation of AOS2-nYFP and WRKY36–cCFP or AOS2-nYFP and PIL15-cCFP led to the reconstitution of GFP signal, whereas no signal was detected when AOS2-nYFP and cCFP were co-expressed. Co-transformation of cCFP and nYFP was used as negative control. (**c**) Co-IP assays indicated the interaction between WRKY36 and AOS2 in plants. 3xFlag-WRKY36 and AOS2-GFP were co-expressed in *N. benthamiana* leaves. The co-expression of 3xFlag-WRKY36 and GFP was used as a negative control. (**d**) RT-qPCR analysis of *AOS2* gene expression levels 0, 24, 48, and 72 h after *R. solani* inoculation. Mock was treated with RNase-free water. *Rs-α-tubulin* was used as the internal control for RT-qPCR. (**e**) RT-qPCR analysis of *SWEET11* gene expression levels 0, 24, 48, and 72 h after *R. solani* inoculation. Mock was treated with RNase-free water. *Ubiquitin* was used as the internal control for RT-qPCR. (**f**) The incidence of disease in leaves treated with mock (RNase free water) or *dsAOS2* after inoculation with *R. solani* at 24, 48, and 72 h. (**g**) The statistical results of % of lesion area covered on the leaves shown were calculated. (**h**) The transient assay showed that WRKY36, PIL15, and AOS2 both activated *SWEET11* expression in an additive manner. The data in (**d**–**f**) are presented as the mean ± SE (n = 3). Statistical differences between groups were analyzed using the Student’s *t*-test (* *p* < 0.05; ** *p* < 0.01; **** *p* < 0.0001; ns indicates nonsignificant). Different letters above the bars indicate significant differences at *p* < 0.05.

Recently, we identified that the *R. solani* effector protein AOS2 interacts with WRKY53 to activate *SWEET2a* and *SWEET3a* for sugar nutrition in pathogens [10]. Since WRKY36 and PIL15 interact with WRKY53, the interaction between AOS2 and WRKY36 as well as AOS2 and PIL15 was assessed. The results of the Y2H assay revealed that AOS2 interacts with WRKY36 and PIL15 (Figure 7a). The BiFC results confirmed that AOS2 interacts with WRKY36 and PIL15 in the nucleus of tobacco cells (Figure 7b). In addition, the Co-IP assay results indicated that 3xFlag-WRKY36 interacts with AOS2-GFP but not with GFP, indicating that WRKY36 interacts with AOS2 (Figure 7c). Spray-induced gene silencing (SIGS) of *AOS2* inhibited *R. solani-*induced *SWEET11* expression [10,42] (Figure 7d,e). To investigate whether AOS2–WRKY36–PIL15 form a transcriptional complex to activate *SWEET11*, the transient assay was performed. The results indicated that AOS2, WRKY36, and PIL15 activate *pSWEET11*, and the co-expression of AOS2–WRKY36–PIL15 revealed higher activation against *pSWEET11* than the expression of AOS2, WRKY36, or PIL15 alone (Figure 7f).

## 3. Discussion

In any infection, the goal of pathogens is to hijack nutrients from the host. Extensive studies revealed that SWEET sugar transporters are the targets of various pathogens [43,44,45]. Pathogenic infection activates *SWEET* expression during the early infection stage to obtain sugars from host plants; therefore, inhibition of *SWEET* activation during pathogen infection could successfully control disease [10,34]. However, SWEET also plays key roles in grain filling. The mutation of *SWEET* genes exhibited defects in grain development [46]. Some studies reported that the mutation of TAL-binding sequences in *SWEET* gene promoters successfully improved the resistance of rice to bacterial blight without any growth defect [47]. However, the TFs that regulate *SWEET* in a tissue-specific manner to balance rice resistance and normal growth have not been studied in detail.

### 3.1. WRKY36–PIL15–AOS2 Activates SWEET11 to Efflux Sugar

**Figure 8:**
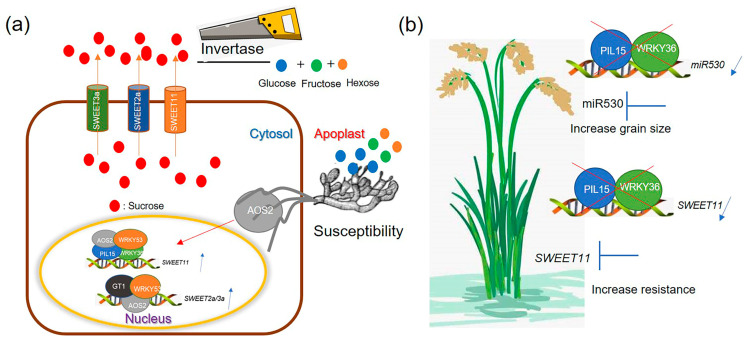
Speculative model shows the PIL15-WRKY36 transcription complex regulates ShB resistance and seed development in rice (Extension from [10]). (**a**) The AOS2 secreted by *Rhizoctonia solani* and WRKY36–PIL15 form a transcription factor complex, activating *SWEET11* in the nucleus. Sucrose is exported from the cytoplasm to the extracellular vesicles through SWEET11, and is metabolized into glucose and fructose through cell wall invertase, which are utilized by *Rhizoctonia solani*. (**b**) The PIL15 and WRKY36 transcription factor complexes negatively regulate rice grain size and resistance to ShB by activating *miR530* and *SWEET11*. If the activations of *miR530* and *SWEET11* by PIL15 and WRKY36 are inhibited, rice yield and ShB resistance are improved.

SWEET11 negatively regulated the resistance of rice to ShB, suggesting that *SWEET11* is a susceptible gene to ShB; however, it positively controlled the grain filling. To assess whether *SWEET11* expression is tissue specific, the Y1H assay was conducted between *SWEET11* and the TFs WRKY36 and PIL15 that regulate resistance to *R. solani*. The Y1H assay revealed that WRKY36 and PIL15 bind to the promoter to activate *SWEET11*. After *R. solani* inoculation, *wrky36* and *pil15* mutants were less susceptible whereas *WRKY36 OXs* and *PIL15 OXs* were more susceptible to ShB. Further genetic combinations revealed that *wrky36/pil15* was less susceptible to ShB than *wrky36* and *pil15*. In addition, overexpression of *SWEET11* in *wrky36* or *pil15* background increased the *wrky36* or *pil15* ShB-resistant phenotype, and the lesion lengths on *SWEET11 OX/wrky36* and *SWEET11 OX/pil15* were similar with on *SWEET11 OX*. These data suggested that WRKY36 and PIL15 might activate *SWEET11* to increase susceptibility of ShB. Furthermore, WRKY36 interacted with PIL15 to form a transcriptional complex, and WRKY36 and PIL15 had synergistic effects on the activation of *SWEET11*. *SWEET11* and *PIL15* expressions are sensitive to *R. solani* inoculation, while *WRKY36* expression level is not altered by *R. solani* infection. However, WRKY36 activates *SWEET11* expression, suggesting that WRKY36 protein might be post-translationally modified upon *R. solani* infection to increase its activity or stability to form more stable transcriptional complex harboring WRKY36–PIL15. *WRKY36 OX* plants exhibited an enlarged lamina joint angle, a typical BR signaling phenotype. Further screening revealed that WRKY36 interacts with WRKY53, a key BR signaling TF. However, *wrky36* mutant is different from *wrky53* mutant [41,48]; it developed normal plant height, suggesting that WRKY36 might has functional redundancy with other WRKY genes for regulating BR signaling. Furthermore, WRKY53 interacted with PIL15; however, *pil15* mutants and *PIL15 OX* plants did not exhibit BR signaling phenotype. However, WRKY36, PIL15, and WRKY53 negatively regulated the resistance of rice to ShB. These data suggested that WRKY36–PIL15–WRKY53 might form a transcriptional complex to regulate defense of rice to ShB. Recently, we demonstrated that AOS2, an effector protein secreted from *R. solani*, interacts with WKRY53 to activate *SWEET2a* and *SWEET3a* to hijack sugar nutrition from rice [10]. Interestingly, AOS2 also interacts with WRKY36 and PIL15 in the nucleus to activate *SWEET11*, and suppression of *AOS2* expression using the SIGS approach inhibited the *R. solani*-infection-mediated induction of *SWEET11*. These data suggested that *R. solani* secrets effector protein AOS2 into the nucleus of a plant cell; further, AOS2–WRKY36–PIL15 transcriptional complex activates sucrose transporter *SWEET11* for obtaining sugars from rice plants (Figure 8a). Since transcription factor complex involving AOS2 activates multiple *SWEETs*, the expression levels of *SWEET2a* and *SWEET3a* in *PIL15* and *WRKY36* mutants and overexpressing plants were detected. The RT-qPCR results showed that the expression of *SWEET2a* and *SWEET3a* in *PIL15* and *WRKY36* mutants and overexpressing plants did not exhibit a certain pattern. In addition, the expression levels of *SWEET11* in *WRKY53* mutants, overexpressing plants, and *gt1* mutant plants were also detected. RT-qPCR results showed that the expression level of *SWEET11* in *WRKY53* overexpressing plants was higher than that in wild-type plants, but there was no difference between *wrky53* mutant and wild-type plants. Meanwhile, there was no difference in the expression level of *SWEET11* between the *gt1* mutant and wild-type plants (Figure S5), implying that *R. solani* effector AOS2 might interact with different rice transcription factors to activate different sets of *SWEETs* for sugar nutrition. These findings further implied the complex regulation of *R. solani* and rice interaction, and surprisingly, a single effector AOS2 could control glucose and sucrose efflux from rice by hijacking different transcriptional complexes.

### 3.2. WRKY36–PIL15–miR530 Signaling Negatively Regulates Grain Size

The *sweet11* mutant exhibited improved resistance to ShB but defective grain filling. WRKY36 and PIL15 activate *SWEET11* to negatively regulate the resistance of rice to ShB; therefore, seed development was examined in the mutants. Unlike *sweet11* mutant, *wrky36* and *pil15* mutants developed larger seeds than wild-type plants. The seed length of *wrky36* mutant was longer but the width was the same compared with wild-type plants. The seed length and width of *pil15* mutant were longer than those of wild-type plants. The seeds of *WRKY36 OX* and *PIL15 OX* were smaller than the wild-type seeds. These developmental features resulted in higher 1000-grain weight of *wrky36* mutants, higher 100-grain weight of *pil15* mutants, lower 1000-grain weight of *WRKY36 OX* and lower 100-grain weight of *PIL15 OX* than that of their corresponding wild-types. The seed setting ration, panicle numbers per plant, and seed numbers per panicle of *wrky36* mutants were similar to the wild-type plants. *WRKY36 OX* plants have fewer seed numbers per panicle compared to the wild-type plants, but the seed setting ration and panicle numbers per plant of *wrky36* mutants were similar to the wild-type plants. The seed setting ration and panicle number per plant of *pil15* mutants *or pil15 RDs* and *PIL15 eGFP OX* or *PIL15 OXs* were similar than wild-type plants. Compared with wild-type plants, *PIL15 eGFP OX* or *PIL15 OXs* have fewer seed numbers per panicle, while *pil15 mutants* or *pil15 RDs* have more seed numbers per panicle, suggesting that WRKY36 and PIL15 are specifically inhibit seed size in rice.

WRKY53 positively regulates seed size by activating BR signaling, suggesting the differential regulation of seed development by WRKY53 and WRKY36. MicroRNA (miRNA) is a type of non-coding small RNA that regulates gene expression by altering the translation efficiency or stability of targeted mRNA. It plays an important role in regulating plant growth, development, and response to pathogens [49,50]. Previously, PIL15 was reported to activate *miR530*, a negative regulator of seed development [24]. Promoter sequence analysis indicated that W-box and G-box appear in the *miR530* promoter. Y1H, transient, and ChIP assays confirmed that WRKY36 bound to *miR530* promoter to activate its transcription. In addition, *miR530* expression level was significantly lower in the developing caryopsis of *wrky36* mutants but higher in *WRKY36 OX* plants, which further confirmed the transcriptional activation of WRKY36 to *miR530* in plants. These data suggested that WRKY36–PIL15–SWEET11 in the green tissues negatively regulated the resistance of rice to ShB, and WRKY36–PIL15–miR530 in the seeds negatively regulated grain size (Figure 8b).

### 3.3. The Potential Application of WRKY36–PIL15 in ShB-Resistant Breeding

Increasing yield and resistance are some of the main goals of crop breeding. However, the signaling pathways of resistance and yield are often antagonistically regulated [18]. Our study demonstrated that WRKY36 and PIL15 activate *SWEET11* and *miR530* in the green tissue and seeds, respectively. *wrky36* and *pil15* mutants exhibited increased resistance to ShB and yield by controlling grain size, respectively. This suggested that *WRKY36* and *PIL15* might be the molecular targets for ShB-resistant rice breeding. *wrky36* and *pil15* mutants exhibited increased yield under the normal condition; however, we believe they will have advantage under *R. solai* infection. Currently, the genome sequence of rice core resources is available, and Crispr/Cas9-mediated genome editing technique is improving fast. Therefore, identification of useful gene sources for resistance and yield trade-off will be helpful for the precise design of molecular breeding. During domestication and improvement, rice evolved genetically to adapt the environmental changes as well as improve yield. In the rice population, SNP and indel (insertion and deletion) occurred in a high frequency, which makes different traits of rice cultivars [51]. In this study, *wrky36* and *pil15* mutants exhibited resistance to ShB and developed large grain; therefore, investigation of elite haplotypes of *WRKY36* and *PIL15* with relatively lower expression levels will provide useful gene source for rice breeding. Integration of those minor effect genes could generate elite cultivars by cross, a general breeding approach in rice. Further, maize sheath blight causes severe yield effects, and it is also caused by *R. solani* AG1-IA, the same pathogen causing rice ShB; and some mechanisms are close between rice and maize [11]. Therefore, the results we identified in rice might be able to be phenocopied in maize, which could help maize breeders to increase efficiency. Lastly, our study provides a useful approach for simultaneously improving rice yield and ShB resistance, and the mechanism could be useful for crop breeding.

## 4. Materials and Methods

### 4.1. Plant Materials and Growth Conditions

Wild-type (WT) (*Oryza sativa* L. Nipponbare, Zhonghua 11, and Longjing 11), *sweet11* mutants (Nip), *PIL15 OXs* (Nip), *pil15 RDs* (Nip), *wrky36* mutants (ZH11), *WRKY36 OXs* (ZH11), *pil15* mutants (ZH11), *PIL15 eGFP OXs* (ZH11), *wrky53* (LJ11), and *WRKY53 OXs* (LJ11) were used in this study. The plants were inoculated with *R. solani* AGI-1A and grown for approximately 2 months in a greenhouse at 30–24 °C (day and night). The tobacco used for injection (*N. benthamiana*) was grown for approximately 1 month in a 22 °C light incubator (16 h day/8 h night).

### 4.2. Pathogens

*R. solani* AG1-IA was cultured on potato dextrose agar (PDA) medium. The 0.4 × 0.8 cm^2^ of wood bark was placed on the edge of a 90 mm PDA medium. Further, 1 cm^2^ of fungal mass was placed at the center. Fungal mass is used for leaf inoculation, while wood bark is used for leaf sheath inoculation. The *Petri dish* was incubated in dark at 30 °C for approximately 2 days. The infected bark was placed at the angle between the penultimate leaf sheath of rice. Image J 1.53e software was used to measure the typical lesion length and lesion area after 1–7 days of inoculation (https://imagej.nih.gov/ij/, accessed on 8 September 2023). Six leaf sheaths or leaves from each line were analyzed, and the experiments were repeated three times. The data in disease spot statistical chart are presented as the mean ± SE (n > 10).

### 4.3. Generation of Transgenic Rice Plants

To generate overexpression lines, *Ubi: WRKY36* and *35S: PIL15* were transformed into ZH11 plant calli. CRISPR/Cas9-generated mutants (*sweet11*, *wrky36*, and *pil15)* were used. *PIL15 OXs* (Nip), *pil15 RD* (Nip), *wrky53* (LJ11), and *WRKY53 OX* (LJ11) were generated as described previously [37,52]. A specific 300-bp fragment from *WRKY36* was cloned into the pH7GWIWG2(II) vector and then transformed into *pil15* to construct *wrky36/pil15* double mutants. *SWEET11* was cloned into the pCambia1381-Ubi vector and then transformed into the *pil15* to construct *SWEET11 OX*/*pil15*. *SWEET11* was cloned into the pCambia1381-Ubi vector and then transformed into the *wrky36* to construct *SWEET11 OX*/*wrky36*.

### 4.4. Yeast-One Hybrid Assay

In the study, all *pHISi-1* carriers were linearized using *Xho*I before transformation. The 2.0 kb of *SWEET11* promoter sequences (*Xho*I site was mutated) and rice cDNA TF library (Ouyibio, Shanghai, China) were cloned into *pHISi-1* and *pGAD424* to obtain *pSWEET11*-pHISi-1 and *pGAD424-TF* yeast vectors, respectively. The *pGAD424-TF* plasmid was used to transform YM4271 yeast strain carrying the linearized *pSWEET11*-pHISi-1. Two DNA fragments containing two adjacent G-box and one normal G-box element in the 2.0 kb of *miR530* promoter were inserted into *pHISi-1* to obtain the *pmiR530* A-pHISi-1 and *pmiR530* B-pHISi-1 plasmids, respectively. Similarly, four fragments predicted on the 2.0 kb of *miR530* promoter, each containing one W-box, were inserted into *pHISi-1* to obtain the *pmiR530* a/b/c/d -pHISi-1 plasmids, respectively. The coding sequences of *PIL15* and *WRKY36* were cloned into the *pGAD424* vector. The recombinant *pGAD424-WRKY36/PIL15* plasmid was used to transform YM4271 yeast strain carrying the linearized *pmiR530* A/B/a/b/c/d-pHISi-1. *pGAD424* empty vector was used as the control. The analysis of the interaction between proteins and DNA was conducted on synthetic dropout (SD) -Leu or -His media containing various concentrations of 3-aminotriazole (3-AT). The relevant primer information is given in Table S1.

### 4.5. Yeast-Two Hybrid Assay

The coding sequences of *WRKY36*, *PIL15*, *WRKY53*, and *AOS2* were cloned into the yeast expression vector *pGAD424* or *pGBT9*. The recombinant *pGAD424-PIL15*/*WRKY36/WRKY53* plasmid was used to transform AH109 yeast strain. The *pGBT9-WRKY36*/*WRKY53/A*OS2 plasmid was used to transform Y187 yeast strain. The diploid yeast cells were grown on SD/-Leu-Trp, -His, or -Leu-Trp-His media containing various concentrations of 3-AT to screen for interactions between proteins after mating. The relevant primer information is given in Table S1.

### 4.6. Co-IP

The coding sequences of *PIL15*, *WRKY36*, *AOS2*, and *WRKY53* were cloned into the *pGD3G3Flag* and *pGD3GGM* vectors, respectively, to generate the *PIL15-3xFlag*, *WRKY36–GFP*, *AOS2-GFP*, *3xFlag-WRKY36* and *3xFlag-WRKY53* plasmids. All plasmids were used to transform *Agrobacterium* GV3101 and expressed in *N. benthamiana* leaves by co-infiltration. The injected leaves were placed in dark for 2 days. Co-IP experiment was conducted as described previously [23]. The relevant primer information is given in Table S1.

### 4.7. BiFC Assay

The *PIL15*, *WRKY36*, and *AOS2* were fused with N-terminus of YFP in *PXNGW*, *PIL15*, *WRKY36*, *WRKY53* were fused with C-terminus of CFP in *PXCGW*. *PIL15–nYFP*, *WRKY36–nYFP*, *AOS2-nYFP*, *WRKY36–cYFP*, *WRKY53–cYFP* [10], and *PIL15–cYFP* were generated. The corresponding vector was transferred into *Agrobacterium tumefaciens* strain GV3101. The strain was grown overnight on LB medium containing appropriate antibiotics at 28 °C. Collect bacteria, resuspend them in buffer (10 mM MES, pH 5.6, 10 mM MgCl_2_, and 150 µM acetosyringone), and then incubate at room temperature for 3 h before inoculation. The tobacco leaves were manually injected. Incubate plants at room temperature under continuous low light for 2 to 4 days [53]. The relevant primer information is given in Table S1.

### 4.8. Microscopic Observation

*A. tumefaciens*-infected *N. benthamiana* leaves were analyzed at 48 h after injection. Leaf discs were placed on a slide and visualized using a X36 objective lens on an inverted microscope (TOKYO Corporation, Tokyo, Japan). GFP and CFP fluorescence were observed using an excitation wavelength of 488 nm, while RFP was observed using an excitation wavelength of 561 nm. When observed under a microscope, both GFP and RFP fluorescence channels are opened. The appearance of green fluorescence indicates the presence of interactions between proteins. H2B-RFP is used as a nuclear localization marker. If GFP and RFP undergo fluorescence merging, it indicates that the protein interaction is in the nucleus.

### 4.9. Transactivation Assay

Effectors *35S: PIL15*, *35S: WRKY36*, reporter *pSWEET11 GUS*, and internal control *35S: LUC* were transformed into protoplasts [54]. The β-glucuronidase (GUS) activity was detected using the GUS assay kit (Promega, Madison, WI, USA). The expression of luciferase (LUC) was detected using a luciferase assay kit (Promega, Madison, WI, USA) [55,56]. The relevant primer information is given in Table S1.

### 4.10. RNA Isolation and RT-qPCR

Total RNA was extracted from rice seedlings or inoculated leaves using RNAiso Plus reagent (Takara, Dalian, China), and genomic DNA was removed. cDNA was synthesized using the PrimeScript RT reagent kit with gDNA Eraser (Takara, Dalian, China) as per the manufacturer’s instructions. RT-qPCR was performed using SYBR qPCR Master Mix (Vazyme, Nanjing, China). The relative expression levels were calculated using the 2^−ΔΔ^CT method [57]. The relevant primer information is given in Table S1. *Ubiquitin* was used as the internal control for RT-qPCR. The data are presented as the mean ± SE (n = 3).

### 4.11. Chromatin Immunoprecipitation (ChIP)-qPCR Assay

According to the reference paper, three-week-old overexpressing rice plants with GFP tags were subjected to chromatin immunoprecipitation. DNA was used for ChIP-qPCR analysis [58]. The relevant primer information is given in Table S1.

### 4.12. SIGS Assay

One-month-old rice was sprayed with enzyme-free water and *dsAOS2*, and inoculated with *R. solani*. Sample and extract RNA from 0 to 72 h after inoculation to detect the expression levels of *AOS2* and *SWEET11*. The relevant primer information is given in Table S1.

### 4.13. Lamina Joint Assay

The 1 cm of the flag leaf, the lamina joint, and 1 cm of the leaf sheath were selected with good growth conditions at the rice heading stage. The angles of lamina joint bending were measured using Image J 1.53e software (https://imagej.nih.gov/ij/, accessed on 8 September 2023) [59].

### 4.14. Production Shape Assay

For the statistical analyses of seed length and width, more than 100 seeds from *WRKY36*, *PIL15*, and *WRKY53* mutants and overexpressions were calculated. For the statistical analyses of plant height, number of effective grains per main panicle, panicle number per plant, and seed number per panicle, the number of statistical samples for *WRKY36* and *PIL15* mutants and overexpressions were 20 plants.

### 4.15. Statistical Analysis

Prism 8 was used for statistical analysis (GraphPad, San Diego, CA, USA). All data were expressed as mean ± standard error. The comparison between different groups was conducted through one-way analysis of variance. Significance was determined using Student’s *t*-test. *p* < 0.05 was considered significant. Significant differences: * *p* < 0.05; ** *p* < 0.01; *** *p* < 0.001; **** *p* < 0.0001 (Student’s *t*-test). Different letters above the bars denote statistically significant differences (*p* < 0.05).

## Data Availability

The original contributions presented in this study are included in the article/Supplementary Material. Further inquiries can be directed to the corresponding authors.

## Supplementary Materials

The following supporting information can be downloaded at: https://www.mdpi.com/article/10.3390/plants14040518/s1, Figure S1. Screening of WRKY36 and PIL15. The expression levels of the 15 putative transcription factors were analysed after 0, 12, 24 and 48 h of *R. solani* infection. Ten leaves were sampled at each time point for RNA extraction. Figure S2. *SWEET11* expression levels in *wrky36* and *pil15* single mutants, *wrky36/pil15* double mutants, *SWEET11 OX*, *SWEET11 OX/pil15* or *SWEET11 OX/wrky36*, and *PIL15 OX* or *WRKY36 OX* plant leaf sheaths by RT-qPCR. (**a**) The expression of *SWEET11* in WT, *pil15*, *wrky36*, and *wrky36/pil15* was examined using RT-qPCR. (**b**) The expression of *SWEET11* in WT, *pil15*, *SWEET11 OX*, and *SWEET11 OX/pil15* was examined using RT-qPCR. (**c**) The expression of *SWEET11* in WT, *wrky36*, *SWEET11 OX*, and *SWEET11 OX/wrky36* was examined using RT-qPCR. (**d**) The expression of *SWEET11* in WT, *PIL15 eGFP OXs*, and *WRKY36 OXs* was examined using RT-qPCR. Statistical differences between groups were analyzed using the Student’s *t*-test (* *p* < 0.05; ** *p* < 0.01; *** *p* < 0.001; **** *p* < 0.0001; ns indicates nonsignificant). Different letters indicate significant differences between groups at *p* < 0.05. Figure S3. *SWEET11* expression level in *wrky36* and *pil15* single mutant seeds by RT-qPCR. (**a**) The expression of *SWEET11* in WT, *wrky36-10*, and *wrky36-11* was examined using RT-qPCR. (**b**) The expression of *SWEET11* in WT, *pil15 RD 2*, and *pil15 RD 22* was examined using RT-qPCR. Statistical differences between groups were analyzed using the Student’s *t*-test (* *p* < 0.05; ** *p* < 0.01; *** *p* < 0.001; **** *p* < 0.0001; ns indicates nonsignificant). Different letters indicate significant differences between groups at *p* < 0.05. Figure S4. Y1H assay of the binding of WRKY53 to the *SWEET11* promoter. The 2.0-kb region of *SWEET11* promoter sequences (*Xho*I site was mutated) and the *WRKY53* coding sequences were cloned into *pHISi-1* and *pGAD424* to obtain *pSWEET11*-pHISi-1 and *pGAD424-WRKY53* yeast vectors, respectively. The *pGAD424-WRKY53* plasmid was used to transform the Y2M4271 yeast strain carrying the linearized *pSWEET11*-pHISi-1. *pGAD424* empty vector was used as control. The analysis of the interaction between proteins and DNA was conducted on synthetic dropout (SD) -Leu or -His media containing different concentrations of 3-aminotriazole (3-AT). The relevant primer information is shown in Table S1. Figure S5. *SWEET11* expression levels in *WRKY53* mutants, overexpressed plants, and *gt1* mutants. *SWEET2a and SWEET3a* expression levels in *WRKY36* and *PIL15* mutants and overexpressed plant leaf sheaths. (**a**) The expression of *SWEET11* in LJ11, *WRKY53 OX*, and *wrky53* was examined using RT-qPCR. (**b**) The expression of *SWEET2a* in WT, *WRKY36 OXs*, and *wrky36* was examined using RT-qPCR. (**c**) The expression of *SWEET3a* in WT, *WRKY36 OXs*, and *wrky36* was examined using RT-qPCR. (**d**) The expression of *SWEET2a* in WT, *PIL15 eGFP OXs*, and *pil15s* was examined using RT-qPCR. (**e**) The expression of *SWEET3a* in WT, *PIL15 eGFP OXs*, and *pil15s* was examined using RT-qPCR. (**f**) The expression of *SWEET3a* in WT, *PIL15 OXs*, and *pil15 RDs* was examined using RT-qPCR. (**g**) The expression of *SWEET2a* in WT, *PIL15 OXs*, and *pil15 RDs* was examined using RT-qPCR. (**h**) The expression of *SWEET11* in WT and *gt1* was examined using RT-qPCR. Statistical differences between groups were analyzed using the Student’s *t*-test (* *p* < 0.05; ** *p* < 0.01; *** *p* < 0.001; **** *p* < 0.0001; ns indicates nonsignificant). Different letters indicate significant differences between groups at *p* < 0.05. Table S1. Primers used in this study.
