# Supplementary material for: WRKY36–PIL15 Transcription Factor Complex Negatively Regulates Sheath Blight Resistance and Seed Development in Rice
Source: Plants (Basel). 2025 Feb 8;14(4):518. doi: 10.3390/plants14040518 (PMC11858971; doi:10.3390/plants14040518)
Supplement: Supplementary file 1 [file plants-14-00518-s001.zip › plants-3425235-supplementary.pdf]

Table S1. Primers used in this study

Primer Name	Sequence (5'-3')
For Yest-two hybrid assay vector construction	
OsWRKY36-F	GAATTCATGTATGCGTGCATGGAAG
OsWRKY36-R	GTCGACTCAGAAGGAGGTGAAGGC
OsPIL15-F	GAATTCATGTCCGACGGCAACGACTT
OsPIL15-R	AGATCTTTATGTTTCAGCCCCATCTCT
OsWRKY53-F	GAATTCATGGCGTCCTCGACGGGGGGG
OsWRKY53-R	GTCGACCTAGCAGAGGAGCGACTCGACGA
RsAOS2-F	GAATTCATGCGTTTCCTACTCTTT
RsAOS2-R	GGATCCTAGCAGGTGAAGGTAGAT
For Bimolecular Fluorescent Complimentary assays plasmid construction	
OsWRKY36-GWF	GGGGACAAGTTTGTACAAAAAAGCAGGCTTCATGTATGCGTGCATGGAAGG
OsWRKY36-GWR	GGGGACCACTTTGTACAAGAAAGCTGGGTGGAAGGAGGTGAAGGCGC
OsPIL15-GWF	GGGGACAAGTTTGTACAAAAAAGCAGGCTTCATGTCCGACGGCAACGACTT
OsPIL15-GWR	GGGGACCACTTTGTACAAGAAAGCTGGGTGTGTTTCAGCCCCATCTCTCTC
OsWRKY53-GWF	GGGGACAAGTTTGTACAAAAAAGCAGGCTTCCTCGAGATGGCGTCCTCGACGGGG
OsWRKY53-GWR	GGGGACCACTTTGTACAAGAAAGCTGGGTGGGGCCCCTAGCAGAGGAGCGACTCG
RsAOS2-GWF	GGGGACAAGTTTGTACAAAAAAGCAGGCTTCATGGCCGACCAAATCGTGCTG
RsAOS2-GWR	GGGGACCACTTTGTACAAGAAAGCTGGGTGGAGTTCATCAAATCTACCCCACTCG
For yeast-one hybrid assay vector construction	
OsWRKY36-F	GAATTCATGTATGCGTGCATGGAAG
OsWRKY36-R	GTCGACTCAGAAGGAGGTGAAGGC
OsPIL15-F	GAATTCATGTCCGACGGCAACGACTT
OsPIL15-R	AGATCTTTATGTTTCAGCCCCATCTCT
OsWRKY53-F	GAATTCATGGCGTCCTCGACGGGGGGG
OsWRKY53-R	GTCGACCTAGCAGAGGAGCGACTCGACGA
OsSWEE11-F	ATATTTTCATAGGGCACTTGCAA
OsSWEE11-R	TGCTACTGGTGATGAAGGTTATT
OsmiR530-A	tctagaACCAACGCACATGGCACATCGCCACGGTTCACATGGGACTTGCACGGCAGGCCAATGGCACGGCGACACGTGCCGGTTTACACGTGGATCAGTTAGCTAGCAACAATGTTGGATATATTGCTAGCTGCTGCAGTACATgaattc
OsmiR530-B	tctagaATGAACAGCTCGGATTTTATTCGTTTTTACTTTGGATATGAAAGGAACTATTGTCGATGGGTTTTGGATTTGACTGTTCAACATTCTGTACGTGTGGCAGCAACACTGCTAGCTAGCTAGCAGCTTCTAAGAGAAACCATCTGTAGTGCACGTGGGAAAATATCTACTACCTCTGTCCCATATTACCTGTCGTATTGAATgaattc
OsmiR530-a	tctagaACCAACGCACATGGCACATCGCCACGGTTCACATGGGACTTGCACGGCAGGCCAATGGCACGGCGACACGTGCCGGTTTACACGTGGATCAGTTAGCTAGCAACAATGTTGGATATATTGCTAGCTGCTGCAGTACATgaattc

OsmiR530-b	tctagaATGAACAGCTCGGATTTTATTCGTTTTTACTTTGGATATGAAAGGAACTATTGTCGATGGGTTTTGGATTTGACTGTTCAACATTCTGTACGTGTGGCAGCAACACTGCTAGCTAGCTAGCAGCTTCTAAGAGAAACCATCTGTAGTGCACGTGGGAAAATATCTACTACCTCTGTCCCATATTACCTGTCGTATTGAATgaattc
OsmiR530-c	tctagaATTACCTGTCGTATTGAATTTTTGTTGGTAATGTTTGATCATTCATCTTATTTAAAAAGTTTTGAAATTATTATTTATTTTGTTTGTGACTTACTTTATTATCAAAAAGTACTTTAAGTATGACTTATTATTTTTTTTATATTTGTACTAGTTTTTCAAAACGAATAGTCAAACGTGTACCCTGCTTTAATACAGCACGTAGCACAATTACCATCTGATCCAATTGGAGACCTACTTAGCCTCATAGCATTATAGTCCTGACTGGTATATCATCAGAAGTGTATGCAgaattc
OsmiR530-d	tctagaATGTACTCTCTTCATTCTCAAATAATTATCACTGTATGTACTATGGCTGTCTTAAGTTTGACCACTATCTAAAAATGACTATAATTTTATGAGTAAAAGGAATATTATTAAATTTAGAGAGAATCAACCCAATTAACGATCTGCCATTAATATTGTCTCTTTCTATAATATATCGACGAGTTTATCGAgaattc
For Co-immunoprecipitation assay vector construction	
OsWRKY36-GFP-F	CTCGAGATGTATGCGTGCATGGAAGG
OsWRKY36-GFP-R	GGGCCCGAAGGAGGTGAAGGCGCA
OsPIL15-3×Flag-F	CTCGAGATGTCCGACGGCAACGACTT
OsPIL15-3×Flag-R	GGGCCCTGTTTCAGCCCCATCTCTCT
3×Flag-OsWRKY53-F	GACATGGCGTGTCCCTCGACGGGGGGG
3×Flag-OsWRKY53-R	GGATCCGCAGAGGAGCGACTCGACGA
For quantitative real-time RT-PCR	
qOsWRKY36-F	ACCAGGAGCGATGTGGATGT
qOsWRKY36-R	CACCATGCGGCAATCCT
qOsPIL15-F	TGGCTCATTTCCCTCATCTC
qOsPIL15-R	ATTCGCTATGCCTTGTTGCT
qOsmiR530Pre-F	AGGAAGATGAAGCAGCAA
qOsmiR530Pre-R	AAGAGACCAGGAACAACAT
qOsexpressed protein-F	GGTGTTTCACTTCTGCTGCTC
qOsexpressed protein-R	TCTTCCTCCATTCCATTAGCC
qOsAP2 domain containing protein-F	TGACAGCACAGTCACAACG
qOsAP2 domain containing protein-R	TCAGATGACGAGGCTACCTTC
qOs1MYB family transcription factor-F	AAGGTCACGCAAGGCAATAC
qOs1MYB family transcription factor-R	CAACAGATCTTCCAGTTCCAAT
qOsCore histone H2A/H2B/H3/H4 domain containing protein-F	CTTGTCTGAACTGAAACGCTCC

qOsCore histone H2A/H2B/H3/H4 domain containing protein-R	AAACTGATTGGCTTGACTGTCC
qOs2MYB family transcription factor-F	ACACCCGGTGAAAGCTTAGC
qOs2MYB family transcription factor-R	GGTCCAATGAGACAGTAGGCAA
qOsnucleic acid binding protein-F	CCTACTCCATGAGCGATAGCA
qOsnucleic acid binding protein-R	CCGTCCTTACTCCCAGAATACA
qOsZOS5-08 - C2H2 zinc finger protein-F	GTCGGCATAAAGCAGTCTCCAC
qOsZOS5-08 - C2H2 zinc finger protein-R	AGCACCATCCCTACTTCAGCTC
qOsDUF260 domain containing protein-F	TGCTCGATCTTGATCGGTATG
qOsDUF260 domain containing protein-R	TTGCTCCGAGCTGAAGTAGG
qOsB3 DNA binding domain containing protein-F	CCTACCAAGCTGAAGGCCAA
qOsB3 DNA binding domain containing protein-R	TCTGGAACTTGGGGTGGAGA
qOssingle myb histone-F	CTACCGCTCCAATGTTGACCTC
qOssingle myb histone-R	CGGATCTTGGCTTCTTAACGAC
qOsbasic helix-loop-helix-F	CCAGCAGCAGATTTGGCAAG
qOsbasic helix-loop-helix-R	AGAAAGATCATCATAGCCACCGT
qOscalmodulin binding protein-F	CGAAATAGAGCAGAGTGC
qOscalmodulin binding protein-R	TGAAGCGGTACAGGATAG
qOsZOS3-07 - C2H2 zinc finger protein-F	TGGTGAGACAGTCAATGAGTGC
qOsZOS3-07 - C2H2 zinc finger protein-R	ATCATGCGAGCGAATCTTATG
qOsSWEET11-F	GGGATTTCTGGCTAGTTTCT
qOsSWEET11-R	CGAGGTAGAGGACGATGTAG
qOsSWEET2a-F	GTGTTCGCTCTAATTGTG
qOsSWEET2a-R	AGAACTCCATATGCGAAG
qOsSWEET3a-F	TTGGTAGTGTTGGTCTAG
qOsSWEET3a-R	AAAGGATCCCTTCCTATG
qOsUbiquitin-F	CAAGATGATCTGCCGCAAATGC
qOsUbiquitin-R	TTTAACCAGTCCATGAACCCG

Figure legends

Siting Wang Figure S1

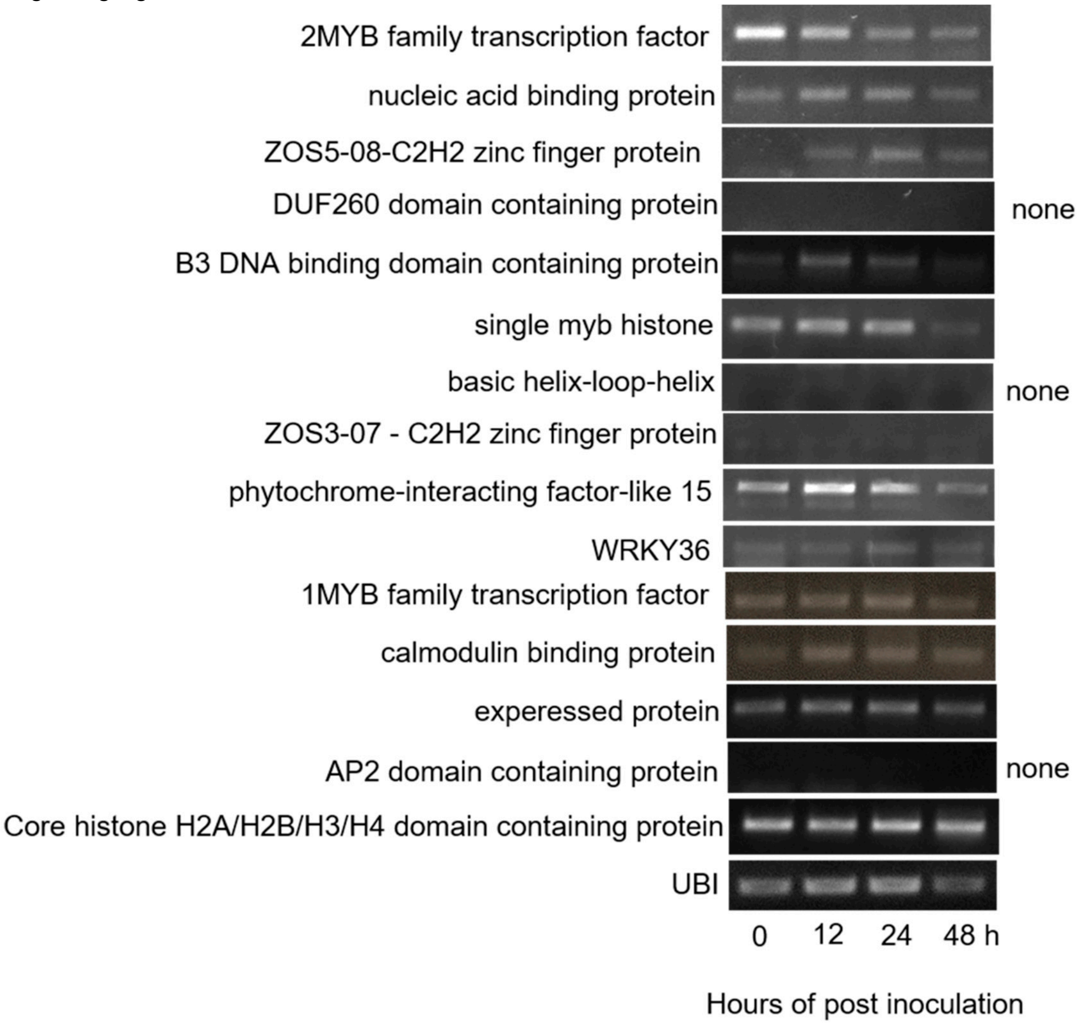

Figure S1. Screening of WRKY36 and PIL15. The expression levels of the 15 putative transcription factors were analysed after 0, 12, 24 and 48 h of *R. solani* infection. Ten leaves were sampled at each time point for RNA extraction.

Siting Wang Figure S2

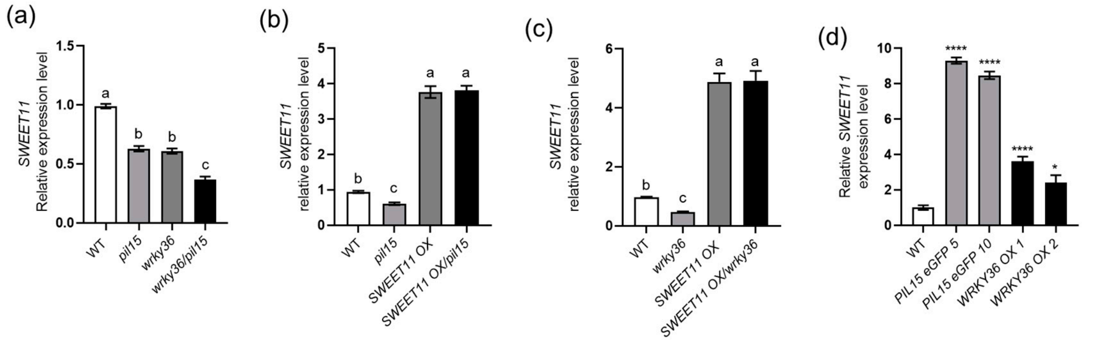

Figure S2. *SWEET11* expression levels in *wrky36* and *pil15* single mutants, *wrky36/pil15* double mutants, *SWEET11 OX*, *SWEET11 OX/pil15* or *SWEET11 OX/wrky36*, and *PIL15 OX* or *WRKY36 OX* plant leaf sheaths by RT-qPCR. (**a**) The expression of *SWEET11* in WT, *pil15*, *wrky36*, and *wrky36/pil15* was examined using RT-qPCR. (**b**) The expression of *SWEET11* in WT, *pil15*, *SWEET11 OX*, and *SWEET11 OX/pil15* was examined using RT-qPCR. (**c**) The expression of *SWEET11* in WT, *wrky36*, *SWEET11 OX*, and *SWEET11 OX/wrky36* was examined using RT-qPCR. (**d**) The expression of *SWEET11* in WT, *PIL15 eGFP OXs*, and *WRKY36 OXs* was examined using RT-qPCR. Different letters indicate significant differences between groups at P<0.05.

Siting Wang Figure S3

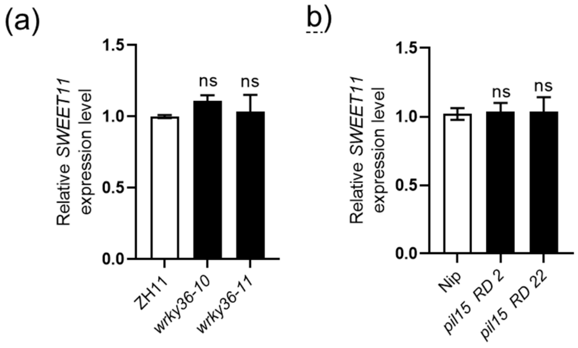

Figure S3. *SWEET11* expression level in *wrky36* and *pil15* single mutant seeds by RT-qPCR. (**a**) The expression of *SWEET11* in WT, *wrky36-10*, and *wrky36-11* was examined using RT-qPCR. (**b**) The expression of *SWEET11* in WT, *pil15 RD 2*, and *pil15 RD 22* was examined using RT-qPCR. Different letters indicate significant differences between groups at P<0.05.

Siting Wang Figure S4

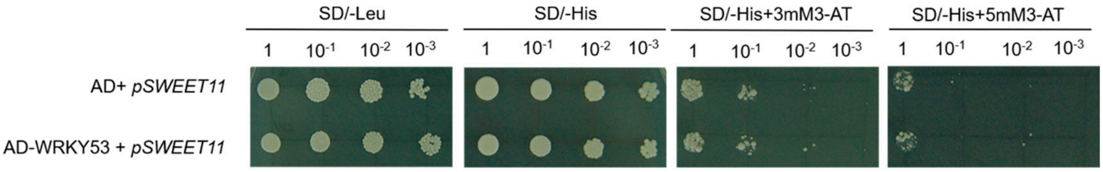

Figure S4. Y1H assay of the binding of WRKY53 to the *SWEET11* promoter. The 2.0-kb region of *SWEET11* promoter sequences (*Xho*I site was mutated) and the *WRKY53* coding sequences were cloned into *pHISi-1* and *pGAD424* to obtain *pSWEET11*-pHISi-1 and *pGAD424-WRKY53* yeast vectors, respectively. The *pGAD424-WRKY53* plasmid was used to transform Y2M4271 yeast strain carrying the linearized *pSWEET11*-pHISi-1. *pGAD424* empty vector was used control. The analysis of the interaction between proteins and DNA were conducted on synthetic dropout (SD) -Leu or -His media containing different concentrations of 3-aminotriazole (3-AT). The relevant primer information is shown in Table S1.

Siting Wang Figure S5

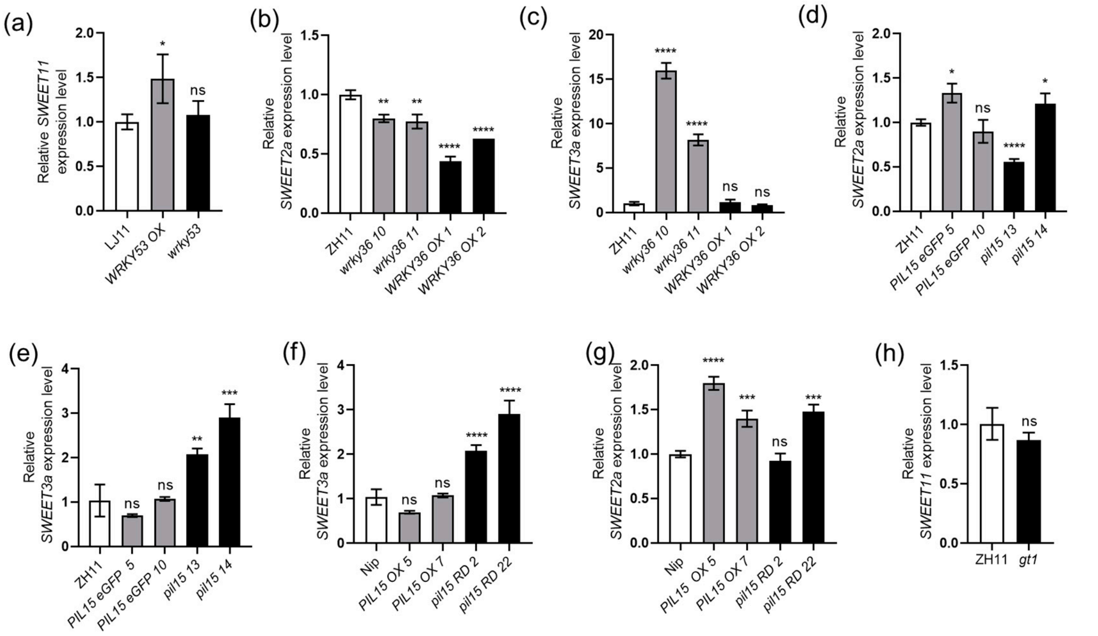

Figure S5. *SWEET11* expression levels in *WRKY53* mutants, overexpressed plants, and *gt1* mutants. *SWEET2a and SWEET3a* expression levels in *WRKY36* and *PIL15* mutants and overexpressed plant leaf sheaths. (**a**) The expression of *SWEET11* in LJ11, *WRKY53 OX*, and *wrky53* was examined using RT-qPCR. (**b**) The expression of *SWEET2a* in WT, *WRKY36 OXs*, and *wrky36* was examined using RT-qPCR. (**c**) The expression of *SWEET3a* in WT, *WRKY36 OXs*, and *wrky36* was examined using RT-qPCR. (**d**) The expression of *SWEET2a* in WT, *PIL15 eGFP OXs*, and *pil15s* was examined using RT-qPCR. (**e**) The expression of *SWEET3a* in WT, *PIL15 eGFP OXs*, and *pil15s* was examined using RT-qPCR. (**f**) The expression of *SWEET3a* in WT, *PIL15 OXs*, and *pil15 RDs* was examined using RT-qPCR. (**g**) The expression of *SWEET2a* in WT, *PIL15 OXs*, and *pil15 RDs* was examined using RT-qPCR. (**h**) The expression of *SWEET11* in WT and *gt1* was examined using RT-qPCR. Different letters indicate significant differences between groups at P<0.05.
